# Comparison of Fatty Acid Binding Protein 3 and Ankle Brachial Index for Predicting Peripheral Artery Disease Outcomes

**DOI:** 10.3390/biom16050735

**Published:** 2026-05-18

**Authors:** Ben Li, Shaima AlQrain, Farah Shaikh, Laszlo Göbölös, Abdelrahman Zamzam, Rawand Abdin, Mohammad Qadura

**Affiliations:** 1 Department of Surgery, University of Toronto, Toronto, ON M5S 1A1, Canada; benx.li@mail.utoronto.ca; 2 Division of Vascular Surgery, St. Michael’s Hospital, Unity Health Toronto, University of Toronto, 30 Bond Street, Suite 7-076, Toronto, ON M5B 1W8, Canada; 3 Institute of Medical Science, University of Toronto, Toronto, ON M5S 1A1, Canada; 4 Temerty Centre for Artificial Intelligence Research and Education in Medicine (T-CAIREM), University of Toronto, Toronto, ON M5S 1A1, Canada; 5 Heart, Vascular, & Thoracic Institute, Cleveland Clinic Abu Dhabi, Abu Dhabi 112412, United Arab Emirates; 6 Department of Medicine, McMaster University, Hamilton, ON L8S 4L8, Canada; 7 Li Ka Shing Knowledge Institute, St. Michael’s Hospital, Unity Health Toronto, University of Toronto, Toronto, ON M5B 1W8, Canada

**Keywords:** fatty acid binding protein 3, ankle-brachial index, prognosis, peripheral artery disease

## Abstract

**Background:** Peripheral artery disease (PAD) impacts more than 200 million individuals globally. Despite its prevalence, management remains suboptimal, partly due to the lack of reliable blood-based biomarkers. The ankle–brachial index (ABI), the current gold-standard test for PAD, is limited by inter-operator variability, misinterpretation, and reduced accuracy in patients with diabetes. Fatty acid binding protein 3 (FABP3) has emerged as a potential biomarker for PAD; however, its prognostic performance relative to ABI remains unclear. This study compared FABP3 and ABI for predicting PAD outcomes using statistical and machine learning approaches. **Methods:** A total of 1001 participants were prospectively recruited, including 644 patients with PAD and 357 without PAD. The primary outcome was 2-year major adverse limb event (MALE), defined as a composite of vascular intervention, major amputation, or acute limb ischemia. At enrollment, plasma FABP3 was quantified using a validated multiplex immunoassay. Kaplan–Meier analysis of MALE-free survival was performed across pre-specified FABP3 tertiles (high [>3.55 ng/mL], moderate [1.55–3.55 ng/mL], and low [<1.55 ng/mL]) and ABI tertiles (severe [<0.40], moderate [0.40–<0.70], and mild [0.70–0.90]), with curve separation assessed using log-rank tests. Multivariable Cox proportional hazards modelling was used to evaluate the independent relationships of FABP3 and ABI with 2-year MALE after adjustment for baseline demographic and clinical covariates. To assess predictive performance for 2-year MALE, an extreme gradient boosting (XGBoost) classification model incorporating 10-fold cross-validation was trained using a combination of clinical covariates, plasma FABP3 levels, and ABI. Discriminatory performance was assessed using the area under the receiver operating characteristic curve (AUC). **Results:** The average participant age was 68 years (SD 12), and 34% (*n* = 340) were women. Mean ABI was 0.75 ± 0.25 and mean FABP3 concentration was 2.97 ± 2.06 ng/mL. Among the 644 participants with PAD, 558 (86.6%) had complete time-to-event data for MALE status, FABP3, and ABI. Over the median follow-up period of 2 years, 140 (25.1%) participants with PAD experienced MALE. Kaplan–Meier analyses demonstrated significant separation in MALE-free survival across FABP3 tertiles (log-rank *p* < 0.001). At 24 months, MALE-free survival was 100.0% in the FABP3 < 1.55 group, compared with 71.1% in the FABP3 1.55–3.55 group and 67.7% in the FABP3 > 3.55 group. In contrast, ABI severity groups showed less pronounced separation, with 24-month MALE-free survival rates of 80.3% for mild ABI, 73.2% for moderate ABI, and 71.3% for severe ABI, without a statistically significant overall difference (*p* = 0.170). In adjusted Cox proportional hazards models, FABP3 demonstrated strong prognostic performance for 2-year MALE. A 1 SD increase in log-transformed FABP3 was independently associated with a higher risk of 2-year MALE (HR 1.90, 95% CI 1.60–2.25; *p* < 0.001), with minimal change after additional adjustment for ABI (HR 1.90, 95% CI 1.60–2.24; *p* < 0.001). Machine learning analyses similarly favored FABP3 over ABI, with the FABP3-based model achieving an AUC of 0.773 compared to 0.686 for the ABI-based model. Adding ABI to the FABP3 model did not improve discrimination. **Conclusions:** Circulating plasma levels of FABP3 are strongly associated with PAD outcomes. Specifically, FABP3 demonstrated a stronger and more robust association with 2-year MALE compared to ABI. This study validates the prognostic value of FABP3 for PAD outcomes in comparison to ABI.

## 1. Introduction

Peripheral artery disease (PAD) impacts more than 200 million individuals globally and arises from progressive atherosclerotic narrowing of the lower limb arteries [1,2]. Although it is a major driver of mortality and limb loss, PAD frequently goes unrecognized and remains inadequately managed [3]. One of the central barriers to improved care is the lack of reliable and standardized biomarkers capable of enabling early identification and prognostication of high-risk patients to inform vascular assessment and management decisions [3].

The ankle–brachial index (ABI) remains the only broadly validated screening modality for PAD; however, its clinical utility is limited by operator dependence, susceptibility to interpretive error, and diminished accuracy in patients with diabetes because of arterial calcification [4,5]. In addition, ABI testing is seldom used in primary care, largely because many general practitioners are often unfamiliar or uncomfortable with performing the procedure and/or interpreting the results [6]. A recent survey reported that nearly four out of five primary care providers do not routinely use ABI, citing barriers such as time limitations, lack of trained staff, and difficulty interpreting findings [7]. Importantly, many clinicians expressed a preference for simpler assessment tools, particularly blood-based tests, which they viewed as more practical and efficient [7]. Accordingly, the identification and validation of a circulating PAD biomarker may enhance clinical management by enabling earlier and more accurate risk stratification, timely initiation of intensive medical therapy, appropriate follow-up, and consideration of revascularization when appropriate.

Fatty acid binding protein 3 (FABP3) is an intracellular molecule typically not detectable in plasma but released into circulation when skeletal muscle is damaged, such as during ischemia caused by PAD [8]. Prior research has linked FABP3 to mitochondrial dysfunction, metabolic syndrome, and various forms of cardiovascular disease [9,10]. Our group has previously shown that increased FABP3 concentrations are associated with both the presence of PAD and greater disease severity [11,12,13]. Although FABP3 is a promising biomarker for PAD, no previous studies have directly compared the prognostic value of FABP3 vs. the gold standard ABI for adverse limb events in patients with PAD, which is important to support clinical implementation [14].

We hypothesized that FABP3 would be a strong predictor of adverse limb events in patients with PAD. Primarily, PAD arises from a complex interplay of metabolic processes, patient characteristics, and clinical factors [15]. Given this multifaceted biology, limb pressure readings based on ABI may not sufficiently capture the pathophysiology of PAD [15]. We hypothesized that integrating a validated PAD biomarker, FABP3, with relevant clinical data would yield comparable prognostic performance compared to the gold standard ABI [15]. In addition, explainable machine learning techniques were used to integrate biomarker and clinical variables, generating prediction models with strong performance and meaningful clinical interpretability [16,17,18]. By enabling timely recognition of high-risk patients through a biomarker-based approach, this strategy has the potential to facilitate earlier initiation of intensive medical/surgical therapy, ultimately improving limb outcomes in this high-risk population.

## 2. Materials and Methods

### 2.1. Ethical Approval

This study received approval from the Unity Health Toronto Research Ethics Board on 8 February 2017 (REB #16-375). Written informed consent was obtained from all participants before study inclusion. All study activities were conducted in accordance with the ethical principles of the Declaration of Helsinki [19].

### 2.2. Study Design

This study was designed as a prospective prognostic investigation, and findings are reported in accordance with the TRIPOD+AI recommendations to ensure transparent reporting of predictive modeling research that incorporates artificial intelligence [20].

### 2.3. Patient Recruitment

Between March 2018 and February 2022, participants were prospectively recruited from outpatient clinics at our hospital, including both patients with PAD and controls without PAD. PAD was defined by an ABI below 0.9 or a toe–brachial index (TBI) below 0.67 in the presence of reduced or absent pedal pulses [21]. Individuals without PAD were required to have an ABI ≥ 0.9, a TBI ≥ 0.67, and normal pedal pulses [21]. Patients were excluded if they had a history of acute limb ischemia, acute coronary syndrome, or elevated troponin levels within the previous three months. Patients with recent cardiac events were excluded to minimize confounding, as FABP3 may also be released following myocardial injury and can correlate with elevated troponin concentrations.

### 2.4. Baseline Patient Characteristics

Baseline demographic and clinical characteristics were collected for all participants, including sex, age, smoking history (current or previous), and comorbidities such as diabetes mellitus, hypertension, dyslipidemia, coronary artery disease (CAD), congestive heart failure (CHF), and previous transient ischemic attack (TIA) or stroke. Cardiovascular risk factors were defined based on American College of Cardiology guidelines [22,23]. Hypertension was defined as diastolic blood pressure ≥ 80 mmHg, systolic blood pressure ≥ 130 mmHg, or use of antihypertensive therapy [22,23]. Dyslipidemia was identified by total cholesterol > 5.2 mmol/L, triglyceride concentrations > 1.7 mmol/L, or treatment with lipid-lowering medications [22,23]. Diabetes mellitus was defined by HbA1c ≥ 6.5% or current use of glucose-lowering therapy [22,23]. Information regarding cardiovascular medication use was also recorded, including statins, acetylsalicylic acid (ASA), angiotensin-converting enzyme inhibitors (ACE-I) or angiotensin receptor blockers (ARB), calcium channel blockers, beta-blockers, loop or thiazide diuretics, oral antihyperglycemic agents, and insulin.

### 2.5. Measurement of Plasma FABP3 Concentrations

At study enrollment, venous blood samples were obtained from the median cubital vein by trained phlebotomists using citrate-containing collection tubes. Plasma was isolated through centrifugation, divided into aliquots, and preserved at −80 °C until analysis, with no interim freeze–thaw exposure. Prior to testing, samples were thawed to room temperature, and FABP3 levels were quantified in duplicate using a commercially available Luminex assay (Bio-Techne, Minneapolis, MN, USA) [24]. The MagPix platform (Luminex Corp., Austin, TX, USA) [25] was calibrated before analysis using Fluidics Verification and Calibration bead kits (Luminex Corp., Austin, TX, USA) [26]. To reduce inter-assay variability, all measurements were completed on a single day. Both intra- and inter-assay coefficients of variation remained below 10%, and a minimum of 50 beads per sample were acquired and analyzed with Luminex xPonent software version 4.3 [27].

### 2.6. Outcomes and Follow-Up

Participants attended outpatient follow-up assessments at 1 and 2 years after the initial evaluation. The primary prognostic endpoint was the occurrence of major adverse limb events (MALE) during the 2-year follow-up interval. MALE was defined as a composite outcome consisting of major amputation above the ankle, vascular reintervention (open surgical or endovascular lower extremity revascularization), or acute limb ischemia, characterized by a sudden reduction in limb perfusion lasting less than 14 days due to arterial thrombosis or embolism.

### 2.7. Model Development and Assessment

For predictive analysis, we utilized Extreme Gradient Boosting (XGBoost), an ensemble-based machine learning technique that combines multiple decision trees to generate a high-performing prediction model [28]. Decision trees operate by recursively partitioning data according to predictor variables to derive outcome-specific classification rules [29]. As a non-parametric algorithm, XGBoost is well suited for large, high-dimensional datasets and offers several advantages, including computational efficiency through parallelization and embedded regularization methods that reduce overfitting and enhance model generalizability [28]. XGBoost was chosen because of its widespread application in both regression and classification studies and its strong performance in predicting clinical outcomes using structured healthcare data [30,31,32].

Participants were randomly allocated into a training dataset (70%) and an independent testing dataset (30%). Using 10-fold cross-validation, the XGBoost classifier was trained to predict 2-year MALE. Predictor variables included ABI and plasma FABP3 levels, together with demographic, clinical, and medication-related factors. Clinical and demographic covariates included CAD, CHF, diabetes, dyslipidemia, hypertension, prior stroke or TIA, smoking history (current and former), age, and sex. Medication variables included ASA, ACE-I or ARB, beta-blockers, calcium channel blockers, loop or thiazide diuretics, oral antihyperglycemic agents, insulin, and statins. Model performance was subsequently evaluated using the independent test dataset, which had not been involved in model training.

### 2.8. Statistical Analysis

Baseline demographic and clinical variables were described using summary statistics. Continuous data are reported as mean ± standard deviation, whereas categorical data are presented as frequencies and percentages. Event rates at 2 years in patients with PAD were reported as numbers and proportions.

Kaplan–Meier survival analyses were conducted in PAD patients with complete MALE time-to-event and baseline FABP3 and ABI data. FABP3 was evaluated using pre-specified plasma concentration thresholds (low [<1.55 ng/mL], moderate [1.55–3.55 ng/mL], and high [>3.55 ng/mL]) as previously defined by our group [33], while ABI was evaluated using established severity categories (mild [0.70–0.90], moderate [0.40–<0.70], and severe [<0.40]) [34]. Kaplan–Meier analysis was performed to estimate MALE-free survival according to FABP3 and ABI categories, and survival distributions were compared using log-rank tests. Pairwise log-rank comparisons were also performed across FABP3 and ABI strata to further characterize between-group differences.

Associations between predictors and MALE-free survival were assessed using Cox proportional hazards regression. FABP3 was modeled as a standardized log-transformed continuous variable (per 1 standard deviation increase in log[FABP3]) for prognostic modeling, and ABI was modeled as a continuous variable scaled per 0.10 increase. Three prespecified multivariable models were evaluated to quantify prognostic performance and incremental value: Model A included clinical covariates plus ABI; Model B included the same clinical covariates plus log-transformed FABP3; and Model C included clinical covariates plus both ABI and log-transformed FABP3. Clinical covariates included sex, age, CAD, CHF, hypertension, dyslipidemia, diabetes, smoking status (current and former), previous stroke/TIA, and relevant medication use, including statins, ASA, beta-blockers, ACE-I or ARB therapy, calcium channel blockers, insulin, oral antihyperglycemic agents, and loop or thiazide diuretics. Incremental prognostic value was assessed using likelihood ratio tests comparing nested models (A vs. C to evaluate the added value of FABP3 beyond ABI; B vs. C to evaluate the added value of ABI beyond FABP3). The purpose of this analytic approach was to directly compare the strengths of the independent associations between FABP3 vs. ABI for 2-year MALE. Model discrimination was assessed using Harrell’s concordance index (C-index). In addition, time-dependent discrimination at 24 months was evaluated using the area under the receiver operating characteristic curve (AUC). Model fit was further compared using the partial Akaike Information Criterion and log-likelihood ratio (LLR) statistics. This analysis provided information regarding the prognostic value of FABP3 vs. ABI for 2-year MALE in the presence of relevant demographic/clinical variables, with a view of assessing clinical utility. All statistical analyses were two-tailed, and statistical significance was defined as *p* < 0.05. Data analysis and model construction were conducted using Python version 3.13.3 [35].

## 3. Results

### 3.1. Patients

The study cohort consisted of 1001 participants. The average age was 68.07 ± 11.62 years, and 340 patients (34.0%) were female. PAD was diagnosed in 644 individuals (64.3%). The mean ABI and TBI were 0.75 ± 0.25 and 0.39 ± 0.18, respectively. Mean plasma FABP3 concentration was 2.97 ± 2.06 ng/mL. Comorbid conditions were common, with 368 participants (36.8%) having diabetes, 334 (33.4%) with CAD, 745 (74.4%) with dyslipidemia, and 725 (72.4%) with hypertension. CHF was present in 43 patients (4.3%), and previous stroke or TIA in 138 (13.8%). Regarding smoking history, 243 participants (24.3%) were current smokers and 495 (49.5%) were former smokers. Medication use included ASA in 546 patients (54.5%), ACE-I or ARB in 545 (54.4%), beta-blockers in 325 (32.5%), calcium channel blockers in 238 (23.8%), loop or thiazide diuretics in 137 (13.7%), insulin in 96 (9.6%), statins in 762 (76.1%), and oral antihyperglycemic agents in 245 (24.5%) (Table 1).

### 3.2. Limb Outcomes

Among the 644 participants with PAD, 558 (86.6%) had complete time-to-event data for MALE status, FABP3, and ABI and were therefore included in the Kaplan–Meier survival analyses. Over a median follow-up period of 24 months, 140 participants experienced MALE, corresponding to an event rate of 25.1%.

### 3.3. Kaplan–Meier Analysis

**Figure 1:**
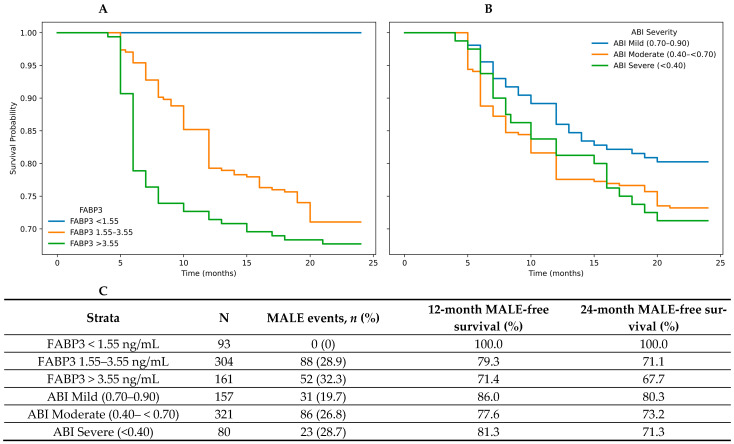
Kaplan–Meier curves for MALE-free survival stratified by FABP3 and ABI levels. Participants were stratified by (**A**) FABP3 using pre-specified plasma concentration thresholds (low [<1.55 ng/mL], moderate [1.55–3.55 ng/mL], and high [>3.55 ng/mL]), while (**B**) ABI was stratified using established severity categories (mild [0.70–0.90], moderate [0.40–<0.70], and severe [<0.40]), with (**C**) MALE-free survival rates reported in tabular format. Abbreviations: MALE (major adverse limb event), FABP3 (fatty acid binding protein 3), ABI (ankle brachial index).

In the survival cohort, participants were stratified by FABP3 levels using pre-specified plasma concentration thresholds (low [<1.55 ng/mL], moderate [1.55–3.55 ng/mL], and high [>3.55 ng/mL]) as previously defined by our group [33], while ABI was evaluated using established severity categories (mild [0.70–0.90], moderate [0.40–<0.70], and severe [<0.40]) [34]. Kaplan–Meier analyses demonstrated significant separation of MALE-free survival across FABP3 strata (log-rank *p* < 0.001). In pairwise log-rank comparisons, FABP3 < 1.55 had significantly higher MALE-free survival than FABP3 1.55–3.55 (*p* < 0.001) and FABP3 > 3.55 (*p* < 0.001). MALE-free survival curves by ABI severity demonstrated less pronounced separation and were not statistically different overall (log-rank *p* = 0.170) (Figure 1).

### 3.4. Cox Regression Analysis

In unadjusted categorical Cox models using FABP3 < 1.55 as the reference, FABP3 1.55–3.55 was associated with an increased hazard of 2-year MALE (HR 3.35, 95% CI 1.69–6.66; *p* = 0.001), as was FABP3 > 3.55 (HR 4.33, 95% CI 2.13–8.78; *p* < 0.001). In unadjusted categorical Cox models, ABI was not significantly associated with 2-year MALE.

In the covariate-adjusted model including FABP3 (Model B), a 1-SD increase in log-transformed FABP3 was associated with an increased hazard of 2-year MALE (HR 1.90, 95% CI 1.60–2.25; *p* < 0.001). This association remained essentially unchanged after additional adjustment for ABI (Model C: HR 1.90, 95% CI 1.60–2.24; *p* < 0.001). ABI (per 0.10-unit increase) was not statistically significantly associated with 2-year MALE in the ABI-only model (Model A; *p* = 0.091) and remained of borderline significance when included with FABP3 (Model C; *p* = 0.051). These results demonstrate that FABP3 is a more robust independent predictor of 2-year MALE compared to the gold standard ABI (Table 2).

### 3.5. Model Predictive Performance

Machine learning model performance metrics consistently favored FABP3 over ABI for predicting 2-year MALE. The ABI-based model (Model A) demonstrated a C-index of 0.668 and a 24-month AUC of 0.686, whereas the FABP3-based model (Model B) demonstrated higher discrimination (C-index 0.753; 24-month AUC 0.773). Adding ABI to FABP3 yielded minimal change in discrimination (Model C: C-index 0.754; 24-month AUC 0.773), while adding FABP3 to ABI substantially improved model fit. Likelihood ratio testing showed that adding FABP3 to the ABI model significantly improved model fit (A vs. C: LLR 55.201; *p* < 0.001), whereas adding ABI to the FABP3 model produced a smaller improvement (B vs. C: LLR 3.799; *p* = 0.051). These results suggest that FABP3 is a stronger and more robust predictor of 2-year MALE compared to the gold-standard ABI.

## 4. Discussion

### 4.1. Key Findings

In the present study, we applied interpretable statistical methods and machine learning approaches to identify FABP3 as a circulating biomarker that is strongly associated with 2-year MALE among patients with PAD. Specifically, we showed a stronger and more robust association between FABP3 and 2-year MALE compared to the gold standard ABI. Integration of FABP3 with demographic and clinical characteristics enabled the development of a highly accurate prognostic model for risk stratification in PAD. Several important findings emerged. First, our cohort exhibited a high prevalence of cardiovascular comorbidities, including hypertension, dyslipidemia, and diabetes, reflecting a representative vascular population and supporting the generalizability of our results. Second, the incidence of 2-year MALE in our cohort was relatively high at 25.1%, demonstrating the need for better prognostication tools to identify high-risk patients for aggressive management to prevent limb loss. Third, Kaplan–Meier analysis demonstrated significantly more pronounced curve separation for FABP3 tertiles compared to ABI tertiles, highlighting that plasma FABP3 levels can better differentiate patients who develop vs. do not develop adverse limb events. Fourth, Cox regression analysis controlling for all baseline characteristics confirmed a significantly stronger independent association between FABP3 and 2-year MALE compared to ABI. Fifth, the XGBoost machine learning model that incorporated both clinical variables and FABP3 achieved excellent predictive performance, with the addition of FABP3 substantially enhancing model accuracy, while the addition of ABI yielded minimal improvement in model performance. Overall, these results suggest that a biomarker-based approach using FABP3 can achieve better prognostic performance compared to the gold standard ABI.

### 4.2. Comparison to Existing Literature

Fatty acid binding proteins (FABPs) have been investigated previously as biomarkers across multiple disease contexts. For instance, Ozawa and colleagues suggested that FABP3 may contribute to the pathogenesis of diabetic nephropathy in murine models [36]. Similarly, Hayashida et al. demonstrated that circulating and urinary FABP3 concentrations may function as early markers of myocardial injury in patients undergoing cardiac surgery [37]. Other investigations have also evaluated FABPs as potential biomarkers in individuals with acute kidney injury requiring renal replacement therapy [38]. Taken together, these findings highlight the growing evidence supporting FABPs as clinically relevant biomarkers for systemic, chronic diseases. Notably, the present study is the first to directly compare the prognostic value of FABP3 vs. the gold standard ABI in patients with PAD, demonstrating a stronger and more robust association between FABP3 and 2-year MALE compared to ABI. These results further establish the prognostic potential of this biomarker for PAD.

Our group has previously demonstrated the potential utility of FABP3 as a biomarker in PAD [11,12,39]. The present study builds on this prior work in several important ways. First, it utilizes the largest and most contemporary cohort to date, comprising over 1000 patients recruited between 2018 and 2022, providing a robust population to validate FABP3’s prognostic utility. Second, to our knowledge, this is the first investigation to directly compare the prognostic performance of plasma FABP3 and ABI for predicting adverse limb outcomes. By demonstrating a stronger association between FABP3 vs. ABI and limb outcomes, we further demonstrate the prognostic value of this biomarker compared to the current clinical gold-standard test for PAD. Third, this study uniquely integrated conventional statistical approaches with advanced machine learning methodologies to assess the prognostic utility of FABP3. Across multiple rigorous analyses, FABP3 consistently enhanced model performance when added to clinical variables, while the addition of ABI yielded minimal improvement in model performance. These results highlight the important contribution of FABP3 to PAD prognostication compared to the gold standard ABI. Collectively, these findings provide strong validation for FABP3 as a blood-based biomarker for PAD, with potential to improve early risk-stratification, ultimately supporting clinical decision-making to reduce the morbidity and mortality associated with this disease.

### 4.3. Explanation of Findings

Multiple biological mechanisms may explain the association between increased FABP3 concentrations and the risk of 2-year MALE. FABP’s are intracellular proteins that facilitate lipid transport [40]. To date, nine distinct isoforms have been identified, all sharing the ability to bind lipid ligands and direct them to cellular sites of metabolism [40]. The precise biological roles of individual FABP isoforms continue to be actively studied [41]. The participation of FABPs in lipid metabolism and transport within key cell populations involved in atherosclerosis, including adipocytes, macrophages, and endothelial cells, suggests an important role in cardiovascular disease pathophysiology [42].

FABP3 is a 14–15 kDa member of the FABP family that is predominantly expressed in cardiac and skeletal muscle tissue [43]. It is primarily located within the cytosol, where it contributes to the cellular uptake and intracellular trafficking of fatty acids [44]. Prior studies have shown that FABP3 is released from skeletal muscle following exercise or injury [8,45,46]. Similarly, patients with PAD experience either intermittent ischemia, as in claudication, or persistent ischemia in chronic limb-threatening disease [47,48], which can lead to the release of FABP3 into the circulation, resulting in elevated plasma levels in individuals with PAD [49,50]. Importantly, the biological pathway related to the synthesis of FABP3 in skeletal and cardiac tissues remains an area of active investigation [14,51,52]. While we have demonstrated the clinical prognostic value of FABP3 for PAD outcomes, further elucidation of the cellular and molecular mechanisms of FABP3 synthesis, transport, and functional activity will be critical to translating this biomarker into effective clinical use [14,51,52].

FABP3 acts as a lipid chaperone that plays a critical role in preserving metabolic homeostasis within cardiac and skeletal muscle tissue [53]. Experimental studies involving FABP3-knockout mice have shown decreased fatty acid uptake, impaired exercise tolerance, increased reliance on glucose metabolism, and subsequent development of cardiac dysfunction [54]. Additionally, FABP3 has been linked to endothelial dysfunction, a condition commonly observed in PAD [55]. Previous research has also implicated FABP3 in metabolic syndrome, mitochondrial dysfunction, and cardiovascular disease [9,10]. In patients with myopathies, skeletal muscle injury, whether exercise-induced or otherwise, triggers the release of FABP3 [45]. PAD can be conceptualized as a form of ischemic myopathy, where repeated tissue ischemia and reperfusion induce skeletal muscle injury [46]. These processes often lead to mitochondrial oxidative stress, enzyme dysfunction, impaired respiration, and ultimately muscle apoptosis [56], with FABP3 released into the circulation as a consequence [57]. The degree of muscle injury may modulate the amount of circulating FABP3, potentially explaining the observed associations between elevated FABP3 levels and poorer limb outcomes [8]. While these pathways provide plausible mechanistic explanations for FABP3 as a prognostic biomarker for PAD, additional basic and translational research is required to confirm these hypotheses.

Notably, we found that FABP3 is associated with 2-year MALE independent of both CAD and diabetes. To reduce potential confounding, patients with recent cardiac events, including acute coronary syndrome or elevated troponin levels within the preceding three months, were excluded from the study. After adjustment for comorbidities such as CAD and diabetes, FABP3 continued to demonstrate an independent association with limb-related outcomes. This is particularly important because diabetes can cause arterial calcification in the lower extremities, often resulting in falsely elevated ABI measurements and limiting the reliability of ABI for PAD prognosis [58]. By showing that plasma FABP3 accurately predicts PAD in both diabetic and non-diabetic patients, we address a key limitation of the current gold-standard test of ABI [58]. Importantly, the ABI was designed primarily as a diagnostic rather than a prognostic tool [59]. Consequently, FABP3 has potential as a biomarker to facilitate broader and more efficient PAD risk-stratification, and complement the use of ABI’s by enabling earlier treatment of high-risk patients to improve limb outcomes.

While several biomarkers including other FABPs have been shown to be associated with PAD outcomes [42,53,60], FABP3 has been consistently demonstrated to be a robust prognostic biomarker for adverse events in patients with PAD [11,12,39]. Thus, it can serve as a significant addition in combination with other biomarkers in a panel format to improve prognostication for PAD outcomes [11,12,13,14,33,42,53,60]. FABP3 was assessed in this study, rather than other FABPs, because of the significant literature supporting its prognostic potential in PAD [11,12,13,14,33]. The advantage of FABP3 over other FABPs is that FABP3 is predominantly expressed in skeletal muscle tissue [43], while other FABPs may be expressed in other tissue types [42,53,60]. Given that adverse limb outcomes in PAD is primarily related to skeletal muscle injury secondary to ischemia, FABP3 may be a particularly sensitive and specific biomarker for PAD prognosis [49,50]. While our work and other studies have demonstrated the prognostic value of FABP3, it would be prudent to continue investigating additional biomarkers including other FABPs in future studies [11,12,13,14,33,42,53,60].

Our machine learning model demonstrated strong predictive performance for several reasons. In contrast to traditional statistical methods, advanced machine learning algorithms are capable of identifying complex, non-linear relationships and interactions within healthcare datasets [61,62], a critical advantage given the multifactorial nature of patient outcomes [63]. Machine learning approaches facilitate automation, detection of complex patterns, and generation of highly accurate predictions, making them particularly valuable for integrating biomarker data, where proteins may interact through intricate biological pathways to influence disease progression [64]. The strong performance of the XGBoost model in this study likely reflects its ensemble-based architecture, which aggregates multiple decision trees to reduce variance, efficiently process large datasets, and limit overfitting [28]. Overall, our findings underscore the value of integrating biomarkers into predictive models, enhancing performance beyond what clinical data alone can provide.

Our study demonstrated a stronger association between FABP3 vs. ABI for 2-year MALE for several potential reasons. Importantly, ABI has been demonstrated to be a relatively poor prognostication tool for limb outcomes in patients with PAD [4,5,7]. This is because ABI can be heavily influenced by calcified vessels in patients with diabetes, which may falsely elevate ABI’s and therefore underestimate limb loss risk in patients with PAD and diabetes [4,5,7]. Given the high prevalence of diabetes in the PAD population, this is a significant limitation [4,5,7]. Additionally, ABI is influenced by operator variability and is subject to inaccurate interpretation, particularly given that many clinicians do not regularly order or interpret ABI’s in generalist settings [4,5,7]. In contrast, FABP3 plasma concentrations can be measured via standard blood tests with reference thresholds and are not significantly influenced by operator dependence [14]. Thus, it may be a more objective test with less variability and a smaller margin for interpretation error compared to ABI [14]. Additionally, ABI is primarily a clinical test based on pressure gradients, while FABP3 incorporates aspects of PAD pathophysiology, enhancing biological relevance and predictive potential [14]. These mechanisms may help explain why FABP3 demonstrated stronger prognostic performance compared with ABI for predicting limb-related outcomes in patients with PAD, further supporting continued investigation of this biomarker for potential integration into clinical practice to improve PAD management and outcomes. Given that PAD is driven by multiple interconnected biological mechanisms and shares many risk factors with other cardiovascular conditions, including CAD and cerebrovascular disease (CVD), our findings support the importance of a multidimensional prognostic approach [65]. The results also reinforce the concept of polyvascular disease, in which clinically relevant atherosclerosis involves several arterial territories simultaneously, highlighting how PAD may increase the likelihood of adverse events in associated conditions such as CAD and CVD [66]. Accordingly, these cardiovascular diseases could similarly benefit from predictive frameworks employed in the present study [66].

### 4.4. Implications

Our findings have several important clinical implications for the management of patients with PAD. Assessment of plasma FABP3 levels may provide clinicians with an additional tool for more precise risk stratification in patients with PAD. Such a biomarker-driven approach may be particularly beneficial in primary care settings, where earlier recognition of high-risk individuals could facilitate timely and targeted intervention strategies [67]. Incorporating FABP3 testing into routine bloodwork can help general practitioners make more informed risk assessments, particularly when access to accredited vascular laboratories for ABI measurements is limited or when interpreting ABI results is challenging [67]. Integrating FABP3 alongside clinical variables within a predictive machine learning framework offers a practical and clinically applicable approach to support decision-making in the management of PAD. The model may be particularly useful for assessing individuals with asymptomatic PAD [68]. Patients with established cardiovascular risk factors and elevated plasma FABP3 levels could undergo additional vascular investigations, such as arterial duplex ultrasonography, to evaluate perfusion and determine PAD severity [69]. Conversely, individuals categorized as low risk may remain under the care of their primary physician, with management focused on cardiovascular risk reduction strategies including statin therapy, ASA, and lifestyle modification [70]. Patients identified as high risk, however, should be referred early to vascular specialists for further evaluation and treatment planning [71]. Among patients assessed by specialists, the predictive model may facilitate individualized, risk-based management decisions. In conjunction with clinical judgment, vascular surgeons may use this tool to help determine the need for: (1) further vascular imaging to define anatomic disease burden and extent [72], (2) initiation of low-dose rivaroxaban therapy [73], and/or (3) consideration of limb salvage interventions [74,75]. Overall, this predictive model may improve PAD management across both primary care and specialty settings by supporting more accurate risk stratification and facilitating earlier therapeutic intervention. Implementation of this approach could help minimize unnecessary specialist referrals, enhance clinical outcomes, and reduce healthcare expenditures [76].

### 4.5. Limitations

Although our findings provide important insights into PAD risk stratification and early therapeutic intervention, several limitations warrant consideration. First, the study was performed at a single academic center, which may reduce the generalizability of the findings to broader and more heterogeneous patient populations. Future multicenter, external validation studies will be important to confirm and extend these findings. Second, machine learning approaches typically benefit from larger datasets, and while our cohort of over 1000 patients is substantial, expanding sample sizes in future research could support the development of more robust predictive models. Finally, the clinical application of FABP3 measurement remains largely confined to research settings. Additional translational research is required to determine the feasibility, cost-effectiveness, and real-world implementation of FABP3 testing within routine PAD care pathways.

## 5. Conclusions

In the present study, we applied interpretable statistical analyses and machine learning techniques to validate FABP3 as a prognostic biomarker significantly associated with 2-year MALE. Importantly, we demonstrated a stronger and more robust association between plasma FABP3 and 2-year MALE compared to the gold-standard ABI. By integrating plasma FABP3 concentrations with relevant clinical characteristics, we developed a robust predictive model capable of accurate PAD prognosis. This FABP3-based framework may improve risk stratification and facilitate individualized management strategies for patients at elevated risk. Earlier identification of high-risk individuals may support timely intervention, including referral to multidisciplinary vascular teams and initiation of targeted medical or surgical therapies aimed at limb preservation. This work highlights the prognostic value of FABP3, which may address the limitations of ABI in predicting PAD outcomes, including unreliability in patients with diabetes, operator dependence, and interpretation error. Furthermore, our results reinforce the need for additional basic science and translational investigations to better define the mechanistic role of FABP3 in PAD development and progression. A deeper understanding of these pathways could advance knowledge of PAD pathophysiology, facilitate the creation of novel personalized therapeutic strategies, and ultimately improve limb outcomes in this vulnerable patient population.

## Figures and Tables

**Table 1:** Baseline characteristics of patients included in the study, including demographics, comorbidities, medications, and other clinical characteristics.

	N = 1001
**Demographics**	
Age, years	68.1 ± 11.6
Female sex	340 (34.0)
**Comorbidities**	
Hypertension	725 (72.4)
Dyslipidemia	745 (74.4)
Diabetes	368 (36.8)
Smoking, past	495 (49.5)
Smoking, current	243 (24.3)
Coronary artery disease	334 (33.4)
Congestive heart failure	43 (4.3)
Previous stroke or transient ischemic attack	138 (13.8)
**Medications**	
Statin	762 (76.1)
ACE-I/ARB	545 (54.4)
ASA	546 (54.5)
Beta blocker	325 (32.5)
Calcium channel blocker	238 (23.8)
Loop or thiazide diuretic	137 (13.7)
Insulin	96 (9.6)
Oral antihyperglycemic	245 (24.5)
**Clinical characteristics**	
PAD	644 (64.3)
Non-PAD	357 (35.7)
ABI	0.75 ± 0.25
TBI	0.39 ± 0.18
Plasma FABP3 level (ng/mL)	2.97 ± 2.06

Continuous variables are expressed as mean ± standard deviation, while categorical variables are summarized as frequency (percentage). Abbreviations: FABP3, fatty acid binding protein 3; PAD, peripheral artery disease; ABI, ankle–brachial index; TBI, toe–brachial index; ASA, acetylsalicylic acid; ACE-I, angiotensin-converting enzyme inhibitor; ARB, angiotensin II receptor blocker; SD, standard deviation.

**Table 2:** Adjusted Cox proportional hazards models assessing associations between FABP3 vs. ABI and 2-year MALE.

Predictor	Model A HR (95% CI)	*p*-Value	Model B HR (95% CI)	*p*-Value	Model C HR (95% CI)	*p*-Value
ABI (per 0.10 increase)	0.91 (0.82–1.01)	0.091	—	—	0.90 (0.81–1.00)	0.051
log (FABP3) (per 1 SD increase)	—	—	1.90 (1.60–2.25)	<0.001	1.90 (1.60–2.24)	<0.001

Model A: Clinical covariates + ABI (per 0.10 increase). Model B: Clinical covariates + FABP3 (per 1 SD increase in log-transformed FABP3). Model C: Clinical covariates + ABI + FABP3. Abbreviations: MALE (major adverse limb event), FABP3 (fatty acid binding protein 3), ABI (ankle brachial index).

## Data Availability

The original contributions presented in the study are included in the article; further inquiries can be directed to the corresponding author.
